# Knockdown of α2,3-Sialyltransferases Impairs Pancreatic Cancer Cell Migration, Invasion and E-selectin-Dependent Adhesion

**DOI:** 10.3390/ijms21176239

**Published:** 2020-08-28

**Authors:** Pedro Enrique Guerrero, Laura Miró, Bin S. Wong, Anna Massaguer, Neus Martínez-Bosch, Rafael de Llorens, Pilar Navarro, Konstantinos Konstantopoulos, Esther Llop, Rosa Peracaula

**Affiliations:** 1 Department of Biology, Biochemistry and Molecular Biology Unit, University of Girona, 17003 Girona, Spain; pedro.guerrero@udg.edu (P.E.G.); laura.miro@udg.edu (L.M.); anna.massaguer@udg.edu (A.M.); rafael.llorens@udg.edu (R.d.L.); 2 Department of Chemical and Biomolecular Engineering, Johns Hopkins University, Baltimore, MD 21218, USA; mbibswong@nus.edu.sg (B.S.W.); konstant@jhu.edu (K.K.); 3 Cancer Research Program, Hospital del Mar Medical Research Institute (IMIM), Unidad Asociada IIBB-CSIC, 08003 Barcelona, Spain; nmartinez@imim.es (N.M.-B.); pnavarro@imim.es (P.N.); 4 Institute of Biomedical Research of Barcelona (IIBB)-CSIC, 08036 Barcelona, Spain; 5 Institut d’Investigacions Biomèdiques August Pi i Sunyer (IDIBAPS), 08036 Barcelona, Spain

**Keywords:** pancreatic ductal adenocarcinoma, α2,3-sialyltransferases, sialyl-Lewis antigens, E-selectin, cell migration

## Abstract

Aberrant sialylation is frequently found in pancreatic ductal adenocarcinoma (PDA). α2,3-Sialyltransferases (α2,3-STs) ST3GAL3 and ST3GAL4 are overexpressed in PDA tissues and are responsible for increased biosynthesis of sialyl-Lewis (sLe) antigens, which play an important role in metastasis. This study addresses the effect of α2,3-STs knockdown on the migratory and invasive phenotype of PDA cells, and on E-selectin-dependent adhesion. Characterization of the cell sialome, the α2,3-STs and fucosyltransferases involved in the biosynthesis of sLe antigens, using a panel of human PDA cells showed differences in the levels of sialylated determinants and α2,3-STs expression, reflecting their phenotypic heterogeneity. Knockdown of ST3GAL3 and ST3GAL4 in BxPC-3 and Capan-1 cells, which expressed moderate to high levels of sLe antigens and α2,3-STs, led to a significant reduction in sLe^x^ and in most cases in sLe^a^, with slight increases in the α2,6-sialic acid content. Moreover, ST3GAL3 and ST3GAL4 downregulation resulted in a significant decrease in cell migration and invasion. Binding and rolling to E-selectin, which represent key steps in metastasis, were also markedly impaired in the α2,3-STs knockdown cells. Our results indicate that inhibition of ST3GAL3 and ST3GAL4 may be a novel strategy to block PDA metastasis, which is one of the reasons for its dismal prognosis.

## 1. Introduction

Pancreatic ductal adenocarcinoma (PDA), the most frequent pancreatic tumor, is considered the one with the direst prognosis among all carcinomas, with the lowest five-year survival rate, of about 5–7% [1]. PDA detection is usually performed at late stages since symptoms are often unnoticed and there is a lack of accurate tumor markers for its detection. In advanced stages, PDA is characterized by its aggressiveness, enhanced by the dense tumor microenvironment that acts as a barrier preventing treatment efficiency [2], and by its ability to spread to other organs at early stages of the disease. In fact, more than 80% of PDA patients are diagnosed with metastatic or unresectable disease [3]. PDA is therefore a challenging disease, and a major knowledge of PDA biology is urgently required to shed light onto new therapeutic and diagnostic strategies.

In recent years, abnormal glycosylation remains in the scientific spotlight since it is a frequent hallmark of cancer and is involved in key steps during cancer formation, progression and metastasis. Glycosylation alterations include changes in sialylation, fucosylation, increased GlcNAc-branching of N-glycans and overexpression of truncated mucin-type O-glycans [4,5,6]. Among these changes, an increase in cell sialyation and in particular of sialyl-Lewis (sLe) antigens, such as sLe^x^ (Neu5Acα2, 3Galβ1,4[Fucα1,3]GlcNAcβ1-R) and sLe^a^ (Neu5Acα2,3Galβ1,3[Fucα1,4]GlcNAcβ1-R), have been shown to be critical to the regulation of cell adhesion, tumor invasion and metastasis [7,8,9,10,11,12]. SLe^x/a^ antigen expression correlates with the aggressiveness and the poor outcome of different tumors [13], such as gastric cancers [14], breast cancer [12], colon cancer [15,16], lung cancer [11] and PDA [17,18]. SLe antigens are ligands of the Ca^2+^ dependent lectin E-selectin, expressed in activated endothelia, and when expressed in cancer cells, they act as mediators of cancer metastasis by favoring selectin-dependent adhesion during the cancer cell extravasation process [19,20].

SLe antigens’ biosynthesis is regulated by a set of glycosyltransferases, namely α1,3/1,4- fucosyltransferases and α2,3-sialyltransferases, two glycoenzymes that are involved in the late steps of glycosylation. Sialyltransferases are a large family of enzymes located in the Golgi, which catalyze the transfer of the sialic acid (SA) through a nucleotide sugar donor CMP-SA. In particular, α2,3-sialyltransferases (α2,3-STs) catalyze the transfer of a SA in α2,3-linkage on β-linked galactose to different carbohydrate acceptors of N-glycans, O-glycans or glycolipids. α2,3-STs comprise of six enzymes (from ST3Gal I to ST3Gal VI), of which ST3Gal III, ST3Gal IV and ST3Gal VI can form the α2,3-SA linkage in mammals on type 1 (Galβ1,3-GlcNAcβ1-R) or type 2 (Galβ1,4-GlcNAcβ1-R) precursor chains, which after their α1,4 or α1,3-fucosylation, will lead to the sLe antigens sLe^a^ or sLe^x^, respectively.

Expression of α2,3-sialyltransferases has been found to be deregulated in malignancy in several types of tumors [21], like colorectal, gastric carcinoma [22,23], breast [24,25,26], ovarian [27], cervix [28] and PDA [10]. Increased mRNA levels of STs are closely related with poor prognosis, reduced overall survival [24,25,29,30] and to cellular resistance to therapies. Previous studies from our group showed that α2,3-STs expression is increased in PDA compared to healthy tissue [10].

Increased expression of type 2 Lewis antigens has been found in PDA tissues when compared with normal pancreas, being sLe^x^ neoexpressed in PDA [31,32]. PDA cell lines also exhibit increased expression of the sLe antigens and the sialyltransferases (STs) involved in their biosynthesis [9,10,33]. Furthermore, the overexpression of either ST3GAL3 or ST3GAL4 in PDA cells increased sLe^x^ expression in their cell membrane glycoconjugates. The overexpressing clones displayed increased adhesion on E-selectin, increased migration on collagen I, a loss of homotypic cell aggregation and a decrease in the overall mouse survival following intrasplenic injection compared to their corresponding controls, which illustrate an aggressive phenotype [9,10,34,35].

Ectopic expression of genes, such as ST3GAL3/4, leads to an artificial cell phenotype of rather limited (patho)physiological significance. To address the role of the α2,3-STs, ST3GAL3 and ST3GAL4 as potential new therapeutic targets of PDA, we have performed a comprehensive analysis of the effects of their knockdown on PDA cell function. We have focused on key steps of cancer progression, such as cell migration and invasion, as well as E-selectin-dependent binding and cell rolling. To this end, we have first assessed the expression of these STs and the sLe antigens in a panel of seven PDA cell lines. ST3GAL3 and ST3GAL4 were knocked down via shRNA in two pancreatic cancer cell lines, BxPC-3 and Capan-1, whose scramble (SC) control cells display high to moderate levels of sLe^x^. The expression of several sialylated determinants on ST3GAL3/4 knockdown cells was characterized by flow cytometry and Western blot (WB). All silenced cells showed a significant reduction in sLe^x^ expression with a concomitant increase in α2,6-sialic acid determinants. Silenced cells displayed reduced migratory and invasive potentials as well as impaired E-selectin binding. Our results demonstrate that the inhibition of these α2,3-STs in cancer, specifically in PDA, hampers cancer progression steps, suggesting that these STs represent new therapeutic targets for PDA.

## 2. Results

### 2.1. Expression of Sialylated Glycan Determinants, α2,3-Sialyltransferases and α1,3/4-Fucosyltransferases in a Panel of PDA Cells

To investigate the contribution of the α2,3-sialyltransferases ST3GAL3 and ST3GAL4 to the adhesive and invasive capabilities of PDA cells, we first characterized their expression levels as well as those of sLe^x^, sLe^a^ and different sialic acid determinants (α2,3 and/or α2,6) in a panel of seven pancreatic cancer cell lines (AsPC-1, BxPC-3, Capan-1, Capan-2, HPAF-II, Panc 10.05 and SW 1990). These cell lines represent varying degrees of pancreatic cancer genetic complexity and different grades of neoplastic differentiation.

**Figure 1:**
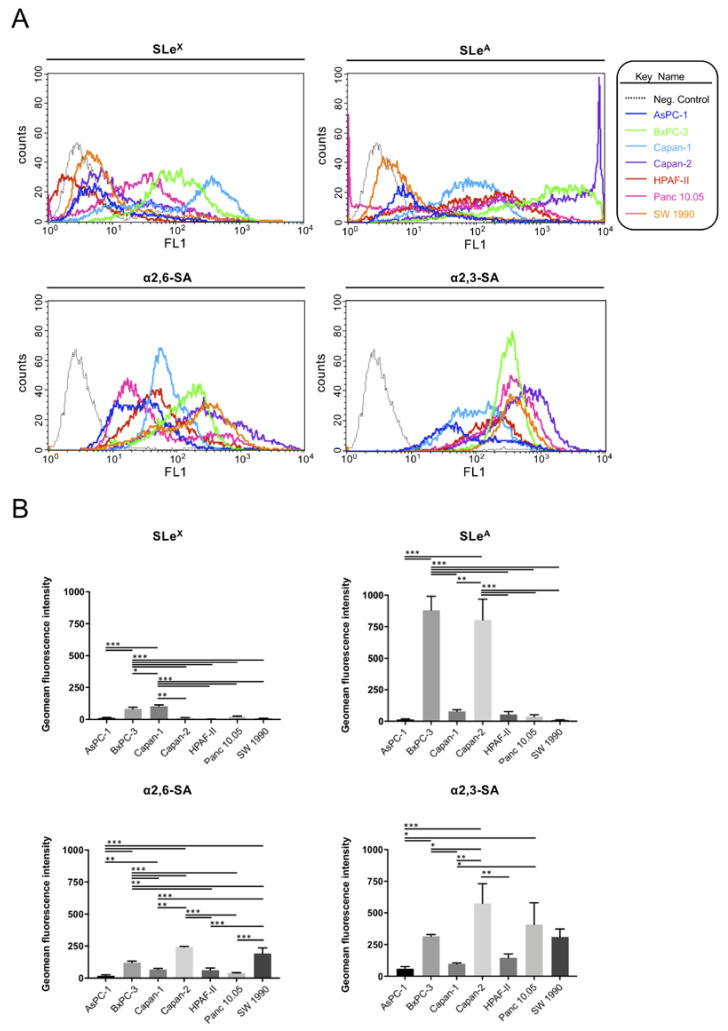
Analysis of the cell surface glycan structures in pancreatic ductal adenocarcinoma (PDA) cell lines by flow cytometry. (**A**): Overlay of the representative cytometry histograms of the different glycan structures of the seven PDA cell lines: sialyl-Lewis x (top left), sialyl-Lewis a (top right), α2,6-sialic acid (bottom left) and α2,3-sialic acid (bottom right). Color legend: Negative control is represented with a continuous dot line: (…), AsPC-1 (dark blue line), BxPC-3 (green line) Capan-1 (light blue line), Capan-2 (purple line), HPAF-II (red line), Panc 10.05 (pink line) and SW 1990 (orange line). (**B**): Geomean fluorescence intensity of the different glycan structures of the seven PDA cell lines: sialyl-Lewis x (top left), sialyl-Lewis a (top right), α2,6-sialic acid (bottom left) and α2,3-sialic acid (bottom right). Data represent mean ± SD from three independent experiments, except for Capan-2, HPAF-II, Panc 10.05 and SW 1990, in which two values were used. ANOVA and Tukey’s multiple comparison post-hoc test was performed. *p* < 0.05: *; *p* < 0.01: ** and *p* < 0.001:***.

Cell surface glycan expression was first analyzed by flow cytometry with specific monoclonal antibodies (mAb) against sLe^x^ and sLe^a^ antigens and lectins: *Sambucus nigra* Lectin (SNA), which binds preferentially to sialic acid attached to terminal galactose in α2,6-linkage, and *Maackia amurensis* Lectin II (MAL II) that binds sialic acid in α2,3-linkage. Flow cytometry experiments with anti-sLe^x^ mAb showed varying expression levels of sLe^x^ and sLe^a^ on the cell surface of the different cell lines (Figure 1A,B top panel). Capan-1 and BxPC-3 cells displayed significantly higher sLe^x^ levels compared with the rest of the PDA cell lines. On the other hand, BxPC-3 and Capan-2 cells had the highest levels of sLe^a^ compared to the rest of the cell lines. Quantitative analyses of the expression of α2,6-sialic acid (SA) determinants using SNA (Figure 1A,B, bottom panel) revealed that Capan-2 and SW 1990 exhibit the highest levels followed by BxPC-3. Analysis of α2,3-SA using MAL II lectin showed that Capan-2 was the cell line with the highest α2,3-SA levels followed by Panc 10.05, BxPC-3 and SW 1990.

**Figure 2:**
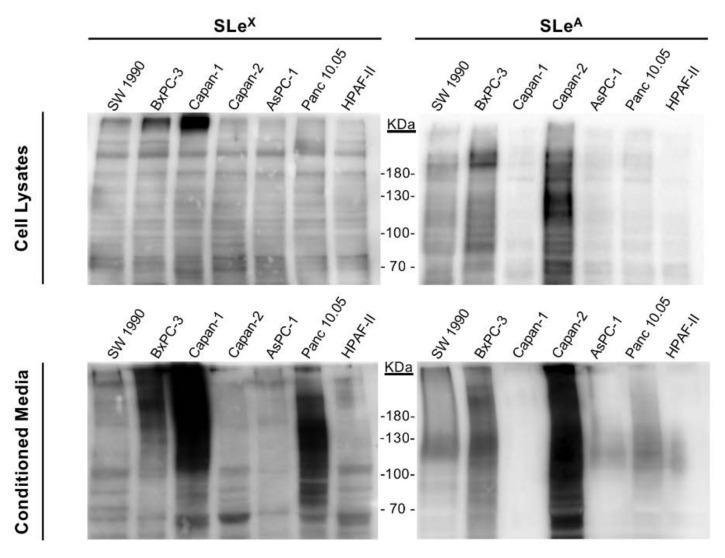
Immunodetection by Western blot of sLe^x^ (left) and sLe^a^ (right) content in proteins from total cell lysates (top) and conditioned media (bottom) of the PDA cells. Blots were probed with clones CSLEX1 mAb against sialyl-Lewis x and the clone 57/27 mAb against sialyl-Lewis a.

The expression of sLe^x^ and sLe^a^ determinants in protein cell lysates and secreted glycoconjugates from conditioned media was analyzed by WB (Figure 2). The results were in line with those obtained for cell membrane glycoconjugates determined by flow cytometry. The highest sLe^x^-expressing cell lines were Capan-1 and BxPC-3, whereas for sLe^a^ were Capan-2 and BxPC-3, in both cell lysates and cell conditioned media, with the signal being higher in secreted glycoproteins of the conditioned media. The main differences of sialylated determinants between cell lines were primarily detected in the high molecular weight region, which could correspond to highly glycosylated mucins, among others [18,32].

**Figure 3:**
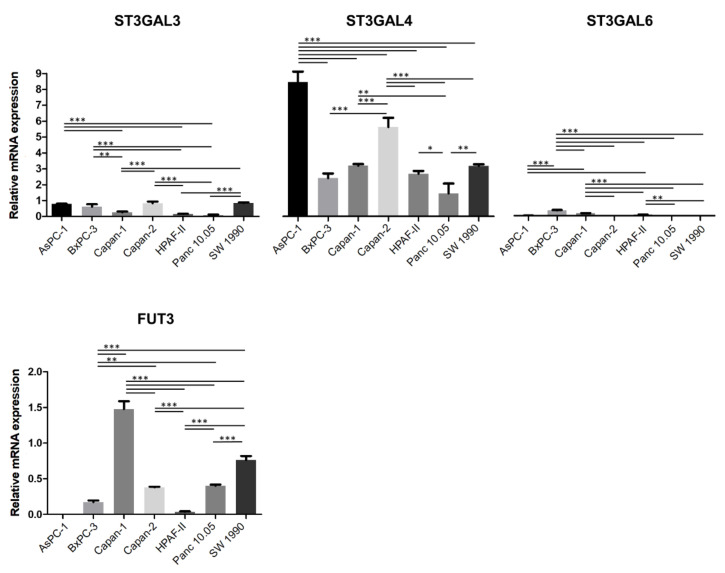
Relative quantification of α2,3-sialyltransferases and α1,3/1,4-fucosyltransfereases. Quantification of ST3GAL3, ST3GAL4, ST3GAL6 and FUT3 mRNA levels by RT-qPCR in the pancreatic cancer cell panel. RNA levels were normalized using TATA-box binding protein (TBP) as a housekeeping gene. Data represent mean ± SD of triplicates of a biological experiment. ANOVA and Tukey’s multiple comparison post-hoc test was performed. *p* < 0.05: *; *p* < 0.01: ** and *p* < 0.001:***.

To identify the most appropriate cell lines to knockdown ST3GAL3 and ST3GAL4, we first determined the mRNA expression levels of the α2,3-ST and the fucosyltransferase genes that code for the enzymes that act in the last steps of SLe antigens’ biosynthesis (Figure 3). Regarding α2,3-ST expression, ST3GAL3, ST3GAL4 and ST3GAL6 mRNA levels were analyzed. ST3GAL6 levels were much lower than ST3GAL3 and ST3GAL4 ones for all cell lines examined in this work. Among all cell lines tested, only Capan-1 and BxPC-3 expressed ST3GAL6 levels above the background. ST3GAL3 expression was also 4–20-fold lower than ST3GAL4 for all cell lines. The cells with the highest ST3GAL3 expression were AsPC-1, BxPC-3, Capan-2 and SW 1990. All cell lines expressed appreciable ST3GAL4 mRNA levels, being AsPC-1, and Capan-2 the cell lines with the highest levels. The expression of α1,3/4-fucosyltransferases, involved in the synthesis of sLe^x^ and sLe^a^ in mammalian cell lines (FUTs-3, -5, -6 and -7) [36], showed that these cell lines exhibited very low levels of FUT5, FUT6 and FUT7, except for Capan-1 cells that displayed much higher FUT6 levels than the rest of the cells (data not shown). FUT3 was the main fucosyltransferase expressed, which was found in six (Capan-1, SW 1990, Panc 10.05, Capan-2, BxPC-3 and HPAF-II) out of the seven cell lines, with Capan-1 expressing the highest levels (Figure 3). AsPC-1 had very low levels, which could not be quantified.

This comprehensive analysis prompted us to select BxPC-3 and Capan-1 cells, which display high sLe^x^ expression and high-to-moderate sLe^a^ expression, for knocking down ST3GAL4 and ST3GAL3 in order to assess the effect of these STs on their biosynthesis as well as their influence on other biologically relevant interactions.

### 2.2. Stable Silencing of ST3GAL4 and ST3GAL3 in BxPC-3 and Capan-1 Cells

To investigate the role of ST3GAL4 and ST3GAL3 in PDA malignant progression, we targeted both genes for silencing through shRNA technology in BxPC-3 and Capan-1 cell lines. Knockdown experiments by inserting the pLKO.1-puro vectors containing the shRNAs against the target genes were first attempted with liposome-based transfection reagents and with specific electroporation kits, but the transfection efficiency for these pancreatic cell lines was very low. Therefore, stable knockdown (KD) of ST3GAL4 and ST3GAL3 genes was achieved by lentiviral delivery of the pLKO.1-puro vector containing the shRNAs against the target genes. PDA cells lines (BxPC-3 and Capan-1) were transduced with five different shRNAs for each target sialyltransferase and the respective sequences were designated as sh-1 to sh-5 for shRNAs against ST3GAL4 and as sh-6 to sh-10 for the ST3GAL3 KD cells. Parental cells were simultaneously transduced with a scramble control containing a non-targeting sequence. No changes in cell morphology or proliferation were observed in the stably transduced cells.

**Figure 4:**
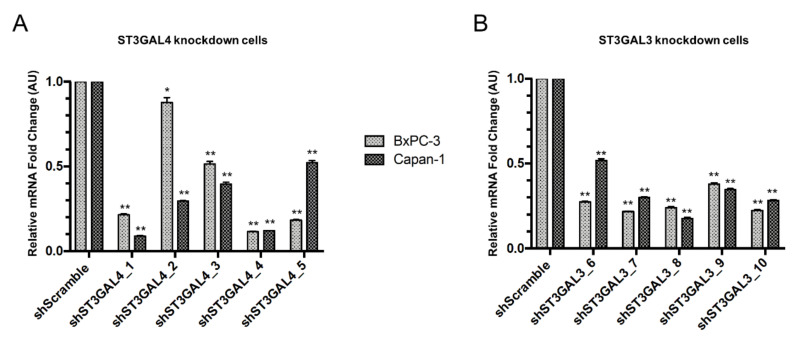
Relative quantification of ST3GAL3 and ST3GAL4 mRNA expression in pancreatic cells after shRNA transfection. (**A**) RT-qPCR validation of the knockdown of ST3GAL4 (shST3GAL4_1-5 cells) in Capan-1 and BxPC-3. Scramble control cells (shScramble) were generated by stable transfection with non-target scramble vector. (**B**) RT-qPCR validation of the knockdown of ST3GAL3 (shST3GAL3_6-10 cells) in Capan-1 and BxPC-3. RNA levels were normalized using TBP as a housekeeping gene. Data are expressed as mean ± SD of three independent experiments * represents *p* < 0.05; **p < 0.01 (Student’s *t* test).

After one-week puromycin selection, ST3GAL4 and ST3GAL3 mRNA expression levels were determined by quantitative real time PCR (RT-qPCR; Figure 4). No changes in ST3GAL4/3 expression levels were detected between parental cells and scramble (SC) cells, therefore SC cells were used as a negative control for expression changes in subsequent experiments. For ST3GAL4 KD cells (Figure 4A), shST3GAL4_1 and shST3GAL4_4 were the ones with the highest knockdown efficiency for Capan-1, showing a decrease of 91 and 87%, respectively. The highest reduction in ST3GAL4 expression in BxPC-3 cells was generated by short hairpins RNAs 1, 4 and 5 with 78%, 88% and 81% decrease, respectively. The five different shRNAs designed to silence ST3GAL3 (Figure 4B) resulted in varying levels of knockdown, all of them greater than 48%. For ST3GAL3 KD cells, shST3GAL3_7 and shST3GAL3_10 showed the highest knockdown efficiencies for BxPC-3 (78% and 77% reduction, respectively) and shST3GAL3_8 and shST3GAL3_10 for Capan-1 (82% and 71%, respectively). To confirm that the knockdown of ST3GAL4 or ST3GAL3 genes did not affect the expression levels of the other ST3GAL genes, we determined the mRNA expression of ST3GAL3, ST3GAL4 and ST3GAL6 genes in all silenced cell lines. As expected, the ST3GAL3 knockdown cells did not show significant changes in ST3GAL4 or ST3GAL6 gene expression. Likewise, the ST3GAL4 knockdown cells did not exhibit significant changes in ST3GAL3 or ST3GAL6 gene expression (data not shown).

### 2.3. Downregulation of ST3GAL4 and ST3GAL3 in BxPC-3 and Capan-1 Cells Reduces sLe^x^ Expression

To explore how the reduction in ST3GAL4 and ST3GAL3 expression levels leads to changes in cell sialylated glycans in BxPC-3 and Capan-1 cells, glycan expression pattern was analyzed by flow cytometry and WB in all ten knockdown cells (sh-1 to sh-10), and their respective controls (SC and parental cells). The knockdown cells that displayed major decreases in sLe antigens are described below and were selected for further in vitro analyses.

**Figure 5:**
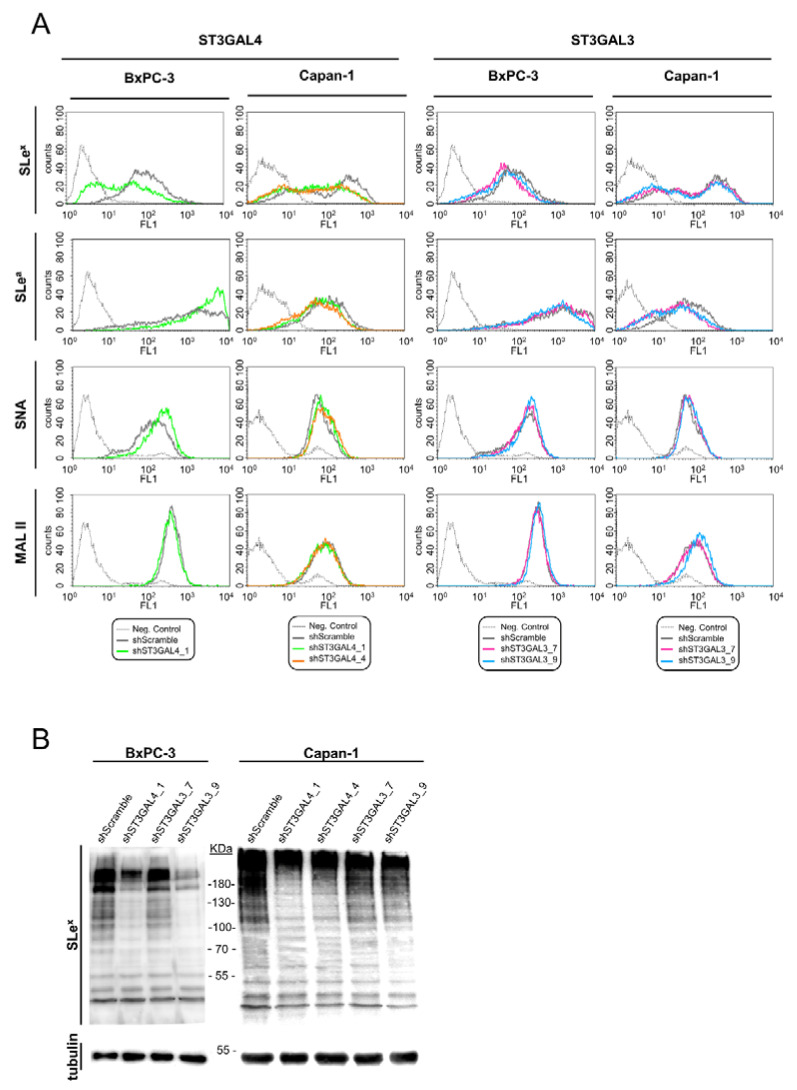
Glycan analysis of ST3GAL3 and ST3GAL4 knockdown BxPC-3 and Capan-1 cells. (**A**) Representative flow cytometry profiles of the cell surface glycans structures detected with sLe^x^ and sLe^a^ antibodies and the lectins SNA and MAL II in knockdown ST3GAL4 cells (left) and ST3GAL3 cells (right) using different shRNA sequences. Negative controls (without the primary antibodies or lectins) are represented using a continuous black dot line: (…), shScramble (black line), shST3GAL4_1 (green line), shST3GAL4_4 (orange line), shST3GAL3_7 (pink line) and shST3GAL3_9 (blue line). (**B**) Immunodetection by WB showing the reduction of sLe^x^ antigens in selected cell lysates from total protein on BxPC-3 (top left) and Capan-1 cells (top right) with anti-sLe^x^ antibody (clone CSLEX1). Anti-tubulin WB of the corresponding membrane is showed on the bottom panel.

In particular, for BxPC-3, shST3GAL4_1 cells were the ones with the most prominent reduction in sLe^x^ membrane expression—up to 68%—when compared to SC median values, as shown by flow cytometry (Figure 5A, Table 1) and WB (Figure 5B). Surprisingly, knockdown of this gene led to a moderate increase of sLe^a^ on cell membrane glycoconjugates detected by flow cytometry (Figure 5A, Table 1), even though we expected either the same or reduced sLe^a^ levels since ST3GAL4 has been reported to also act on type 1 structures [9]. To confirm these results, CA19-9 levels, which correspond to the sLe^a^ antigen, of the total cell lysates were quantified using the mAb 1116-NS-19-9. The levels of CA19-9 in shST3GAL4_1 BxPC-3 cells were indeed increased by 30% relative to the ones of SC BxPC-3 cells.

Lectins’ analyses of shST3GAL4_1 BxPC-3 cells also showed an increase of 53% in α2,6-sialic acid using SNA (Figure 5A, Table 1), suggesting a multiple enzymatic competition of α2,3- and α2,6-STs for type 2 chains. This is in agreement with our previous work showing that increased levels of sLe^x^ are accompanied by decreased α2,6-sialic acid content of membrane glycoconjugates [9]. MAL II analysis did not reveal any significant differences in the total amount of α2,3-sialic acid content (Figure 5A). Sialic acid determinants of protein lysates from the selected silenced cells were also tested by WB using SNA and MAL II lectins. The changes detected in the specific glycan determinants showed the same tendency as the ones obtained by flow cytometry and were found mainly in high molecular weight glycoproteins (data not shown).

For Capan-1, ST3GAL4 KD cells also showed a significant (63–64%) reduction in sLe^x^ content accompanied by a decrease of sLe^a^ levels (in a 32% and 45%; Figure 5A,B Table 2), suggesting that ST3GAL4 is also involved in sLe^a^ biosynthesis in Capan-1 cells as previously described [10]. In accord with BxPC-3 cells, a significantly increased signal in α2,6-SA content was additionally detected in shST3GAL4_1 and shST3GAL4_4 Capan-1 (35 and 24% in the median values compared with the SC cells; Figure 5A, Table 2). No significant changes in MAL II detected glycans were found either by flow cytometry or WB.

For BxPC-3, shST3GAL3_7 and shST3GAL3_9 cells displayed a 33–37% reduction in sLe^x^ relative to SC cells (Figure 5A, Table 1), as also shown by WB of their cell lysates (Figure 5B). ST3GAL3 KD also led to a modest reduction of sLe^a^, in the shST3GAL3_9 cells (Figure 5A, Table 1). Because of the high sLe^a^ content of BxPC-3 cells and to overcome the possible saturation in the immunodetection by immunofluorescence, CA19-9 content was also determined by the quantitative immunoassay and showed a 28% reduction in the shST3GAL3_7 and a 78% reduction in the shST3GAL3_9 cells. Regarding α2,6-SA content, flow cytometry results indicated a significant increase up to 42% for shST3GAL3_9 cells (Figure 5A, Table 1). Both ST3GAL3 KD cells showed also a slight increase trend in α2,3-SA content in flow cytometry analyses using MAL II lectin (Figure 5A). This result could be explained in part because MAL II lectin is unable to bind to sLe^x^/sLe^a^ structures.

For Capan-1, shST3GAL3_7 and shST3GAL3_9 cells also displayed significant reduced sLe^x^ levels (61% and 73%, respectively; Figure 5A, Table 2), which was corroborated by WB of protein cell lysates, which revealed that the changes were mainly found in high molecular weight glycoproteins (Figure 5B). Reduction of ST3GAL3 also led to a significant decrease in sLe^a^ antigen around 40–50% (Figure 5A, Table 2). Regarding the expression of α2,6-SA, a modest increase trend was detected using SNA (Figure 5A, Table 2). MAL II analyses by flow cytometry did not show differences among clones except for a significant increase (39%) in shST3GAL3_9 cells (Figure 5A), which was also confirmed by WB of the protein cell lysates.

Overall, the knockdown of ST3GAL4 and ST3GAL3 significantly decreased sLe^x^ levels to a similar degree for all Capan-1 knockdown cells, whereas in the case of BxPC-3, the reduction of sLe^x^ expression was more pronounced for the ST3GAL4 KD cells (Table 1; Table 2). ST3Gal III enzyme is mostly related with the addition of α2,3-sialic acid upon terminal galactose on type 1 chains (sLe^a^ precursor), while ST3Gal IV plays the major role in the synthesis of sialylated type 2 chains (sLe^x^ precursor). However, our results show the important role of ST3GAL3 in the sLe^x^ biosynthesis, too. The decrease in sLe^x^ was more pronounced in Capan-1 ST3GAL3 knockdown cells than in the corresponding BxPC-3 ones. This difference between cell lines could be explained by the lower expression levels of this gene in Capan-1 compared to BxPC-3.

A significant reduction of sLe^a^ was observed in most of the ST3GAL3 knockdown cells, being also more pronounced for Capan-1 cells (Table 1 and Table 2). ST3GAL4 knockdown also resulted in a decrease of sLe^a^ but only in the Capan-1 cells. BxPC-3, exhibiting very high levels of sLe^a^, did not show a decrease in their corresponding ST3GAL4 knockdown cells, but an unexpected, albeit moderate increase. The reduction in the α2,3-sialylated Lewis antigens was compensated by an increase of α2,6-sialylated structures, which can be explained by the competition of the α2,6-STs for the same substrates that the α2,3-STs. Next, we evaluated the behavior of these knockdown cells, which showed the greatest reduction in sLe^x^ antigens, in cell adhesion and migration in vitro.

### 2.4. ST3GAL4 and ST3GAL3 Knockdown in BxPC-3 and Capan-1 Impaired Pancreatic Cancer Cell Migration

Sialyltransferases have been described to play an important role in mediating migration and dissemination events in diverse cancer cells. In addition, sLe antigens overexpressed on cell membrane glycoconjugates enhance and modulate a wide variety of pathologically relevant processes in cancer, including migration [9,10]. To determine whether the reduction of those sialylated antigens had a phenotypic effect on PDA cells, we have characterized the role in cell adhesion and migration events of the ST3GAL4 and ST3GAL3 KD cells with reduced levels of sLe^x^ on cell surface glycoconjugates.

**Figure 6:**
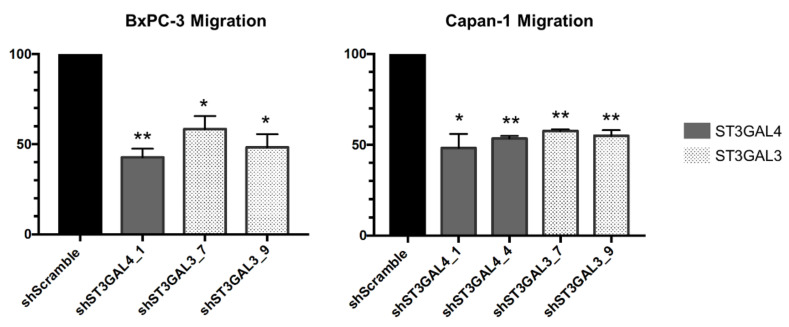
SLe^x^ reduction resulted in impaired cell migration. Relative quantification of cell migration over shScramble cells of BxPC-3 knockdown cells (shST3GAL4_1, shST3GAL3_7 and shST3GAL3_9) (left) and Capan-1 cells (shST3GAL4_1, shST3GAL4_4, shST3GAL3_7 and shST3GAL3_9) (right). Cells were allowed to migrate through 8 µm-pores-type 1-collagen coated Boyden chambers. Migrated cells were fixed and counted. Results are presented as mean ± SEM of three independent experiments. ANOVA and Tukey’s multiple comparison post-hoc test was performed. * *p* < 0.05, ** *p* <0.01.

ST3GAL4 KD cells (shST3GAL4_1 for BxPC-3 and shST3GAL4_1 and shST3GAL4_4 for Capan-1), and ST3GAL3 KD cells (shST3GAL3_7 and shST3GAL3_9, for both BxPC-3 and Capan-1) were allowed to migrate in modified Boyden chambers using FBS as a chemoattractant. Downregulation of α2,3-STs significantly suppressed cancer cell migration in PDA cells in comparison with SC cells for both cell lines, in decrease percentages that ranged from 41% to 57% for BxPC-3 and from 42% to 51% for Capan-1 cells (Figure 6).

To assess whether sLe^x^ reduction of the ST knockdown cells correlates with decreased cell migration, SC cells were incubated with the anti-sLe^x^ mAb CSLEX1 prior to their seeding. Both treated BxPC-3 and Capan-1 SC cells showed a reduction in migration, up to 59% in BxPC-3, and up to 71% in Capan-1 (data not shown). In SC Capan-1 cells treated with anti-sLe^X^ antibody, the decrease in migration was notably higher compared to their corresponding silenced clones. The marked decrease in cell migration in the α2,3-ST knockdown cells as well as in in the SC cells treated with anti-sLe^x^ antibody indicates that sLe^x^ plays a major part in this process.

**Figure 7:**
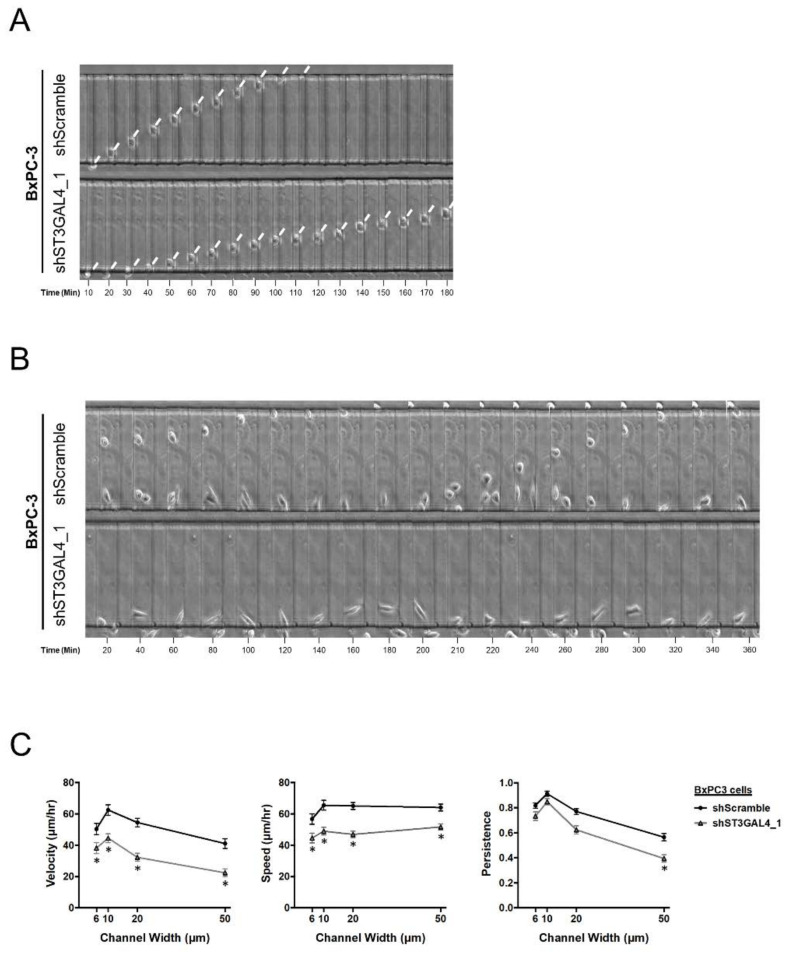
ST3GAL4 knockdown in BxPC-3 cells impaired cell migration inside microchannels. (**A**) Representative time lapse images depicting migration through 10 μm-wide confined microchannels at 10-min intervals of BxPC-3 shScramble cells (top row) and shST3GAL4_1 BxPC-3 cells (bottom row). Arrowheads indicate the leading edge of a migrating cell. (**B**) Illustrative picture showing differences in cell persistence between BxPC-3 shScramble cells (top row) and shST3GAL4_1 BxPC-3 cells (bottom row) migrating inside 50 μm-wide unconfined microchannels in 20-min time intervals. (**C**) Migration velocity (left panel), speed (middle panel) and persistence (right panel) of scramble control and shST3GAL4_1 BxPC-3 cells in PDMS-based microchannels of 10 μm in height, 200 μm in length, and either 6, 10, 20 or 50 μm in width. Data represent the mean ± SEM from at least 3 independent experiments. Dunnett’s multiple comparisons test was used to compare each of the shRNAs to scramble in migration microdevices * *p* < 0.05.

The role of α2,3-sialyltransferases knockdown in cell migration was also assessed inside microchannels of different dimensions. In vivo, disseminated tumor cells migrate within 3D extracellular matrix (ECM) and through longitudinal tracks of prescribed dimensions ranging from 3 to 30 μm in width created by various anatomical structures [37,38,39]. By using a microfluidic device consisting of an array of parallel microchannels of different widths (*W =* 6–50 µm) and prescribed height (*H* = 10 µm) and length (*L* = 200 µm) coated with collagen type I [40,41,42], we characterized the migratory potential of SC and KD BxPC-3 and Capan-1 cells. shST3GAL4_1 BxPC-3 cells, which display the highest reduction in sLe^x^, also showed markedly suppressed migration in all channels (Figure 7). These results demonstrate that ST3GAL4 knockdown reduces BxPC-3 cell motility in a microfluidic system, which mimics aspects of the in vivo microenvironment.

### 2.5. ST3GAL4 and ST3GAL3 Knockdown Reduced Cell Invasion In Vitro

**Figure 8:**
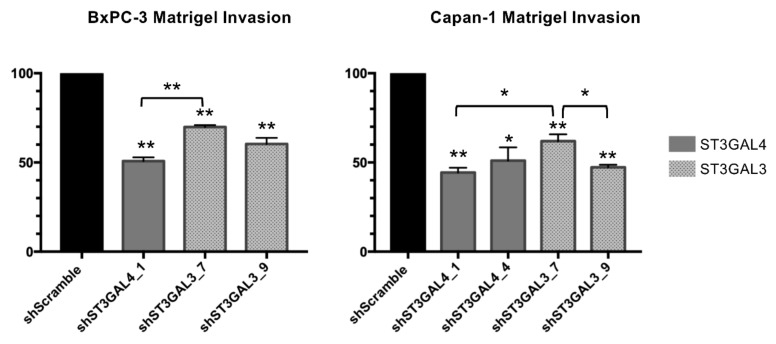
SLe^x^ reduction impaired cell Matrigel invasion in vitro. Tumor cell invasion was evaluated using Boyden chambers in 24-well plates with Matrigel coated inserts. Invading capacity was assessed by quantifying the number of cells that invade in BxPC-3 knockdown (left) and Capan-1 knockdown cells (right) relative to scramble control cells. Data represent the mean ± SEM from at least 3 independent experiments. ANOVA and Tukey’s multiple comparison post-hoc test was performed. * *p* < 0.05, ** *p* <0.01.

To determine whether the decrease in ST3GAL4 and ST3GAL3 expression and their concomitant reduction in sLe^x^ also impaired the invasion of pancreatic cancer cells, transwell invasion assays were performed by coating chambers with Matrigel. ST3GAL4 was more effective than ST3GAL3 knockdown (49% for shST3GAL4_1 versus 30% for shST3GAL3_7) in reducing the invasive potential of BxPC-3 knockdown cells relative to SC cells (Figure 8). This difference between ST3GAL4 and ST3GAL3 depleted BxPC-3 cells could be attributed to the significantly higher reduction of sLe^x^ in the former ones (68% vs. 33–37%; Table 1). Along these lines, ST3GAL4 or ST3GAL3 knockdown diminished Capan-1 cell invasion to a roughly similar level (Figure 8), which is in line with their equivalent reduction in sLe^x^ expression.

To validate the role of sialylation in cell invasion in vitro, we used a blocking antibody against sLe^x^ antigens. Treatment of SC Capan-1 and BxPC-3 cells with anti sLe^x^ antibody impaired invasion by 39% and 55%, respectively (data not shown). Altogether, these results indicate a positive link between the expression of sLe^x^ expression and the invasive capability of PDA cells.

### 2.6. Reduced Levels of sLe^x^ in ST3GAL4 and ST3GAL3 Knockdown Cells Led to Decreased Binding to E-Selectin

**Figure 9:**
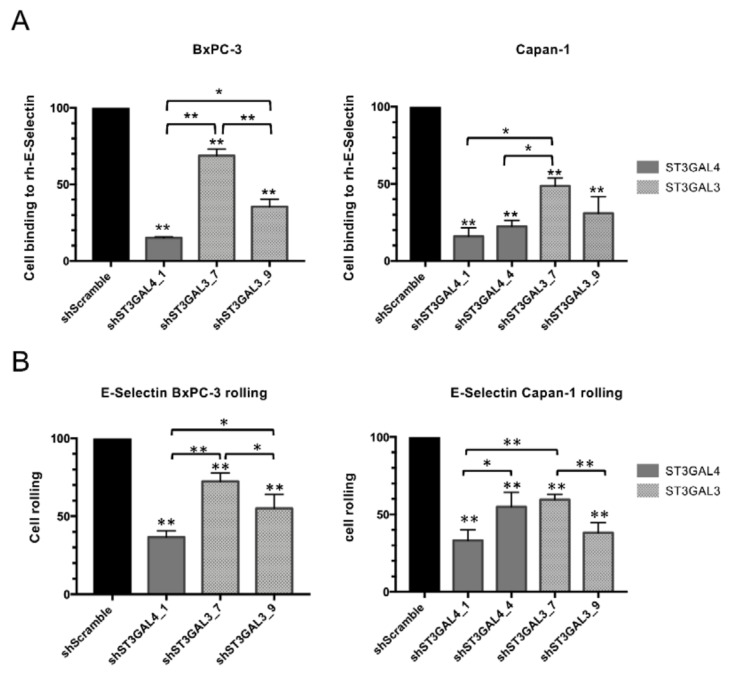
ST3GAL3 and ST3GAL4 knockdown inhibited cell adhesion to rh-E-selectin under static conditions and also impaired cell binding to rh-E-selectin under flow. (**A**) BxPC-3 (left) and Capan-1 cells (right) were incubated over E-selectin coated 96-well plates and allowed to adhere at 37 °C. Adherent cells were quantified after 1 h incubation with MTS-based colorimetric assay. Experiments were performed in triplicate. Results are presented as mean ± SEM of adherent cells respect shScramble cells of three independent experiments. ANOVA and Tukey’s multiple comparison post-hoc test was performed * *p*< 0.05, ** *p* <0.01. (**B**) Reduction in BxPC-3 (left) and Capan-1 (right) knockdown cells binding to rh-E-selectin in comparison to SC control cells flowing over immobilized E-selectin. Graphs depict means and SD from at least three independent experiments.

E-selectin expressed on activated endothelial cells binds to sialylated ligands on the surface of circulating tumor cells slowing them down, which is a key step prior to their extravasation from the circulatory system. We have investigated the impact of ST3GAL4 and ST3GAL3 KD on E-selectin-dependent binding and rolling using BxPC-3 and Capan-1 cells as models. As a first step, we measured cell binding to recombinant human E-selectin (rh-E-selectin) under static (no flow) conditions (Figure 9A). All KD cells displayed significantly decreased adhesion to rh-E-selectin compared to their respective BxPC-3 and Capan-1 SC cells, which is attributed to the reduction of sLe antigens’ levels (Table 1 and Table 2). In BxPC-3 cells, the higher decrease of ST3GAL4 than ST3GAL3 KD cell binding to E-selectin (84% reduction for shST3GAL4_1 vs. 31% and 64% reduction for shST3GAL3_7 and shST3GAL3_9, respectively) was due to the higher reduction in sLe^x^ levels compared to their respective SC cells. In Capan-1 cells, shST3GAL4_1, shST3GAL4_4 and shST3GAL3_9 exhibited similarly reduced levels in E-selectin adhesion and sLe^x^ expression. shST3GAL3_7 was an outlier since the less efficient reduction of E-selectin-dependent binding did not correlate with the extent of decrease in sLe^x^ expression. Nevertheless, the importance of sialylated antigens in E-selectin binding was further demonstrated upon incubation of SC BxPC-3 and Capan-1 cells with an anti-sLe^x^ mAb, which resulted in >80% inhibition. Taken together, these findings reveal that sLe^x^ is critical to the binding of PDA cells to human E-selectin in vitro.

To further investigate the functional importance of ST3GAL4 and ST3GAL3 in the binding of pancreatic tumor cells to E-selectin, we also analyzed this adhesive interaction under dynamic flow conditions using a microfluidic system [43]. Using this system, we perfused SC and KD BxPC-3 and Capan-1 cells under a shear stress level of 1.1 dyn/cm^2^ over immobilized E-selectin (Figure 9B). SC cells interacted more efficiently than KD cells for both cell lines. In line with static assays, ST3GAL4 knockdown BxPC-3 cells showed the highest inhibition to tethering and rolling on E-selectin. Along these lines, ST3GAL4 and ST3GAL3 knockdown cells displayed reduced binding to E-selectin even though moderate differences were noted among different KD Capan-1 cell lines.

## 3. Discussion

Aberrant glycosylation of glycoconjugates on the tumor cell surface facilitates distinct steps of the metastatic cascade of events [4,5,6,20]. In this work, we have addressed the impact of the downregulation of the α2,3-sialyltransferases ST3GAL3 and ST3GAL4 (which regulate sLe^x^ and sLe^a^ biosynthesis) on migration, invasion and E-selectin-dependent adhesion of pancreatic cancer cells.

### 3.1. Diversity in Sialyl-Lewis Antigens’ and their Corresponding Glycogenes’ Expression in PDA Cells

The expression levels of the α2,3-sialyltransferases and α1,3/1,4-fucosytransferases involved in the biosynthesis of sLe^x^ and sLe^a^ in mammals as well as the levels of these sialylayted antigens on cell surface, in cell lysates and conditioned media were evaluated in seven pancreatic cancer cell lines of different genetic complexity that cover the wide tumor heterogeneity that can be found in PDA. In all PDA cells, ST3GAL4 was more abundant than ST3GAL3 (from 4 to 20-fold mRNA increase depending on the cell line). These results were qualitatively in accord with the higher expression levels of ST3GAL4 compared to ST3GAL3 described in pancreatic adenocarcinoma tissues, where ST3GAL4 and ST3GAL3 levels were higher than the mean of pancreatic control tissue by about 4-fold and 2-fold, respectively [10]. Similar results on higher ST3GAL4 relative to ST3GAL3 expression have also been reported in gastric carcinoma [44]. ST3GAL6 was present at near background levels, and could only be detected in Capan-1 and BxPC-3 cells albeit at much lower levels than for ST3GAL3, which is in agreement with our previous data [33].

The expression levels of the α1,3/α1,4-fucosyltransferases (FUT-3, 5, 6, 7 and 9) that catalyze the transference of fucose residues to the N-acetylglucosamine (GlcNAc) on the α2,3-sialylated type 1 and type 2 precursors were also analyzed. We found that FUT3 was the only α1,3/α1,4-fucosyltransferase, which presented noticeable expression levels in most of the cell lines, being Capan-1 the one with highest level, in agreement with recent published data, which found FUT3 between the top-upregulated genes in the aggressive pancreatic cancer cell line Capan-1 [45].

The levels of sLe^x^ and sLe^a^ epitopes on the glycoconjugates of the seven PDA cells were variable among them as expected by their different α2,3-sialyltransferases’ and α1,3/1,4-fucosytransferases’ expression. The differences in sLe^x^ levels between cell lines could be explained by the combination of the several expressed α1,3/α1,4-FUTs and α2,3-STs on the available type 2 precursor chains. AsPC-1 and HPAF-II showed almost undetectable levels of sLe^x^ and sLe^a^ antigens probably due to the very low levels of α1,3/α1,4-FUT, FUT3, whereas on the other hand Capan-1 that showed the highest sLe^x^ levels was the one with the highest levels of FUT3 and FUT6. SNA and MAL II analysis also showed different expression levels of α2,3 and α2,6-sialic acid determinants’ expression among these seven PDA cell lines. The wide diversity in glycan expression of these different PDA cell lines is a reflection of their different phenotype and metastatic potential in agreement with the results obtained from the analyses of N-glycan determinants of several PDA cells [46].

From the seven PDA cell lines, Capan-1 and BxPC-3 were the ones chosen to knockdown α2,3-STs (ST3GAL4 and ST3GAL3) because they presented the highest levels of sLe^x^ on their cell surface, in total protein lysates and conditioned media, and also showed high to moderate levels of sLe^a^.

### 3.2. ST3GAL4 and ST3GAL3 Knockdown Effects on sLe^x^/sLe^a^ Cell Levels

A significant reduction of sLe^x^ was shown in both ST3GAL4 and ST3GAL3 KD cells, in similar percentages for Capan-1 cells while for BxPC-3 cells, the decrease in sLe^x^, was significantly higher for the ST3GAL4 KD cells than for ST3GAL3 ones, reinforcing the role of ST3GAL4 in sLe^x^ biosynthesis as previously described [47]. However, ST3GAL3, which encodes the main enzyme involved in the synthesis of α2,3-sialylated type 1 structures that lead to sLe^a^ biosynthesis, has also a key role in the sLe^x^ biosynthesis in both PDA cell models. These results are also in agreement with other studies on pancreatic, gastric and breast cancer cells where the overexpression of ST3GAL3 led also to an increase of sLe^x^ antigen [9,26,48].

Regarding the changes in sLe^a^ levels, both ST3GAL4 and ST3GAL3 KD Capan-1 cells showed a decrease in sLe^a^ expression. For BxPC-3 cells, the decrease in sLe^a^ was only detected in one of the ST3GAL3 KD cells (shST3GAL3_9), which displayed higher decrease in their E-selectin dependent adhesion than the shST3GAL3_7 cells that did not show a reduction in sLe^a^. ST3GAL3 codifies for the enzyme that preferentially acts on the type 1 structures over type 2 structures, while ST3GAL4 is more active on type 2 and type 3 structures than on type 1 structures [49], which explains the decrease in sLe^a^ in the ST3GAL3 silenced cells.

Reduction of the α2,3 sialylated-Lewis antigens in the knockdown cells also led to an increase in α2,6-sialic acid, which can be explained through enzymatic competition of α2,3 and α2,6-STs for type 1 and 2 chains, determined by SNA, in agreement with our previous work [9].

### 3.3. ST3GAL4 and ST3GAL3 Silencing Effects on Migration and Invasion Capabilities of the Tumor Cells

KD of either ST3GAL4 or ST3GAL3 suppressed the migratory (42–57%) and invasive propensities (33–67%) in both BxPC-3 and Capan-1. The inhibition of motility and invasion was due to decreased expression of sLe^x^ antigen as substantiated by the use of an antibody against sLe^x^. These results are in agreement with previous studies showing that the increase of sLe^x^ via ST3GAL4 or ST3GAL3 overexpression in different carcinomas such as pancreas, gastric or breast leads to an increased invasive phenotype [9,10,26,48]. ST3GAL4 KD have also been reported to decrease the ability of cancer cells to adhere to selectins [47] and to invade and migrate in vitro in gastric cancer [50]. Recently, KD of FUT3 in Capan-1 cells, which is involved in the linking of terminal fucose monosaccharides in α1,3 and α1,4-linkage in the latest steps of sLe^x^ and sLe^a^ respectively, resulted in a high reduction in colony formation and migration as assessed by wound closure assays relative to SC cells [45].

The potential mechanism, underlying the increased invasive phenotype in overexpressed ST3GAL4 in gastric cancer cells, which is accompanied by a concomitant increase in sLe^x^, could involve the activation of tyrosine kinase receptors such as c-Met and its associated downstream signaling effectors [7]. This activation has been postulated to be initiated by secreted and membrane glycoproteins carrying sLe^x^. In this regard, we have also shown that an increase in sLe^x^ in pancreatic cancer cells overexpressing ST3GAL3 alters the glycosylation pattern and modulates the function of important cell membrane glycoproteins, such as α2β1 integrin and E-cadherin, involved in tumor cell adhesion and invasion mechanisms [34]. Altogether, these studies provide evidence for the importance of the sialic acid determinants, in particular of sLe^x^ in the tumor cell progression steps.

### 3.4. ST3GAL4 and ST3GAL3 Silencing Effects on E-selectin Binding of Tumor Cells

Metastasis is a multistep process, which is initiated by the dissemination of cancerous cells from a primary tumor, and involves migration until the development of secondary tumors in a distant organ. In between, cancerous cells have to extravasate the endothelium in the blood vessels, being sialyl-Lewis antigens crucial ligands involved in the initial steps of rolling and arresting of the tumor cells on the activated endothelial cells from blood vessels [21].

In this work, we demonstrated significant impaired binding to human recombinant E-selectin of the ST3GAL4 and ST3GAL3 KD BxPC-3 and Capan-1 cells. In general, higher reduction in E-selectin binding was found in the ST3GAL4 KD cells compared to ST3GAL3 silenced cells in both cell models using microplates and microfluidic assays. These results suggest the key involvement of sialylated structures, and in particular of sLe^x^, in the adhesion to E-selectin, involved in the initial steps of pancreatic cancer cell arrestment on endothelial cells. In agreement with this, the overexpression of FUT1, which competes for the same substrate that α2,3-STs, in BxPC-3 pancreatic cancer cells resulted in a decrease in sLe^x^ levels, which was associated with a reduction in E-selectin-expressing CHO cells binding and an impaired metastatic potential into xenograft transplantation [51,52]. This mechanism is also shared with other neoplasms like in human lung carcinoma cells, in which the up-regulation of FUT3 by TNF-α resulted in an increase of sLe^x^ expression, which mediated enhanced invasion and E-selectin binding [53]. Studies with metastatic prostate cancer cells also showed an increase in the adhesion to E-selectin and in migration and invasion after cell treatment with TNF-α, which led to an increase of sLe^x^ [54]. A further cell treatment with an antibody against sLe^x^ compromised the prostate cell migration and invasion with similar results to those obtained in our cell models.

The importance of targeting the sialyltransferases involved in the late steps of sLe^x^/sLe^a^ biosynthesis has also been shown in other carcinomas such as in lung cancer. In this regard, Yoshihama et al. [8] also reported that the KD of ST3GAL4 in lung metastatic H1299 cells inhibit sLe^x^ expression reducing cell adhesion to HUVEC cells, which suggests its potential as a target for cell metastasis. In ovarian cancer, ST3GAL3 KD sensitized ovarian cancer cells to cisplatin and paclitaxel induced apoptosis [55,56]. Intriguingly, the induction of epithelial-mesenchymal transition (EMT) in colon cancer led to the upregulation of FUT3, ST3GAL3 and ST3GAL4, which are implicated in the final steps of the biosynthesis of sLe^x/a^, suggesting a significant link between those Lewis antigens and EMT in colon carcinoma [57]. Further investigations are necessary to increase our understanding about the influence of sialytransferase KD in EMT process. We have herein shown that the KD of ST3GAL4 and ST3GAL3 confers a partial reduction but not a complete abrogation of the sialyl-Lewis antigens (sLe^x^/sLe^a^), which is in line with another report using HL-60 leukocytic cells [47]. However, the effects of such reduction were enough to downregulate in vitro some of the malignant properties of PDA cells. Blockade of sialic acid using a cell permeable sialyltransferase inhibitor has shown to reduce significantly the synthesis of sialoglycans both α2,3 and α2,6, and it has diminished the adhesive and migratory capability of melanoma cells [58] and suppressed tumor growth by enhancing T-cell mediated tumor immunity [59]. Another sialyltransferase inhibitor, Soyasaponin I (SsaI), that targets α2,3-STs, in particular ST3Gal I, inhibited tumor cell migration and dissemination in the in vivo mouse model with transplanted ovarian cancer cells [27]. Furthermore, this inhibitor targeted ST3Gal IV in breast cancer cells and modified their invasive behavior.

Overall, we have shown that STs, and in particular ST3GAL4 and ST3GAL3 that lead to the sLe^x/a^ biosynthesis can be considered as potential therapeutic targets against pancreatic cancer. This opens a new window to the investigation of compounds that inhibit the corresponding enzymes to block sLe^a^ and neo-expressed sLe^x^ in pancreatic tumor cells to avoid or reduce their metastasis to other organs. The main limitation of this work is the absence of models other than cells. Experiments with animal model systems are needed to corroborate the results described using the human PDA cell lines in order to improve the impact of these studies.

## 4. Materials and Methods

### 4.1. Cell Lines

Human pancreatic cancer cell lines Capan-1, Capan-2, HPAF-II, Panc 10.05 and SW 1990 were purchased from the American Type Culture Collection (ATCC, Manassas, VA, USA). AsPC-1 and BxPC-3 were obtained from the cancer cell repository at Hospital del Mar Medical Research Institute (IMIM) Barcelona, Spain. Cells were cultured at 37 °C in a humidified atmosphere of 5% CO_2_. HPAF-II were cultured in EMEM (Lonza, Walkersville, MD, USA), supplemented with 10% heat-inactivated fetal bovine serum (FBS, Gibco, Life Technologies Corporation, Grand Island NY, USA), 100 U/mL penicillin, 100 U/mL streptomycin and 2 mM L-glutamine (Gibco). Panc 10.05 cells were grown in RPMI-1640 (Lonza) with FBS to a final concentration of 15% and 10 U/mL of human recombinant insulin. AsPC-1, BxPC-3, Capan-1, Capan-2 and SW 1990 Cells were routinely grown in DMEM (Gibco) supplemented with 10% FBS (20% FBS for Capan-1 cells). Routine tests were performed to confirm the absence of mycoplasma.

### 4.2. Conditioned Media, Protein Lysates and Western Blot Analysis

To obtain conditioned media, human pancreatic cancer cells, AsPC-1, BxPC-3, Capan-1, Capan-2, HPAF-II, Panc 10.05 and SW 1990 were cultured in 125 cm^2^ flasks. Once cells reached 80% confluency, they were thoroughly washed and left in serum-free media. After 48 h, media was collected and filtered with a 25 mm diameter sterile syringe filter of polyethersulfone membrane of 0.22 µm pore size (Pall, Ann Arbor, MI, USA). Filtered conditioned media was concentrated to a final volume of 300–400 µL by centrifugation with Amicon^®^ Ultra-15 centrifugal filters of 10 K, previously passivated with 5% Brij-35 overnight. Total protein concentration was determined by QuickStart^TM^ Bradford protein assay (Bio-Rad, Hercules, CA, USA) using bovine serum albumin (BSA) standard (Bio-Rad).

Total protein extracts were obtained mechanically, lysing the cells after 10 min incubation with cold RIPA buffer (50 mM Tris-HCl pH 7.4, 0.1% NP-40, 0.5% sodium deoxycholate, 0.1% SDS, 150 mM NaCl, 2 mM EDTA, 50 mM sodium fluoride, 1 mM PMSF, 0.2 mM sodium orthovanadate and complete ULTRA tablets (protease inhibitors cocktail tablets, Roche, Mannheim, Germany)). Cell lysates were centrifuged and supernatants were collected for their subsequent quantification by Bradford assay as described above.

Western blot (WB) analysis was performed as previously described [60]. Briefly, 50 µg of total protein were loaded into polyacrylamide gels under reducing conditions. The antibodies and solutions used for the immunoblotting are listed below. Blocking buffers: 2% polyvinylpyrrolidone (PVP) in TBST for blocking lectin WB, 3% BSA in TBST for Lewis antigens and 5% powder low-fat milk for tubulin detection. Primary antibodies or lectins: (a) Anti-sialyl-Lewis x (clone CSLEX1, BD Biosciences, San Jose, CA, USA); (b) anti-α-Tubulin (B-7, Santa Cruz Biotechnology, Dallas, TX, USA); (c) Anti-sialyl-Lewis a, (clone 57/27); (d) Biotinylated Sambucus Nigra Lectin (Vector Laboratories, Burlingame, CA, USA) and (e) Biotinylated Maackia Amurensis Lectin II (Vector Laboratories). For the chemiluminescence detection the following peroxidase-conjugated secondary reagents were used: (a) Peroxidase-Conjugated AffiniPure Goat Anti-Mouse IgG + IgM (Jackson immune Research, West Grove, PA, USA); (b) Peroxidase-Conjugate Goat Anti-Mouse IgG (Merck-Millipore, Darmstadt, Germany) and (c) Streptavidin-HRP Conjugate (GE Healthcare, Little Chalfont, UK).

### 4.3. Lentiviral Generation, Viral Transduction and Silencing by Short Hairpin RNA (shRNA)

For shRNA lentiviral infections, five pLKO-1 vectors targeting ST3GAL3: sh-6 (TRCN0000232797), sh-7 (TRCN0000232799), sh-8 (TRCN0000232798), sh-9 (TRCN0000035715) and sh-10 (TRCN0000035716), five vectors targeting ST3GAL4:(sh-1 (TRCN0000005574), sh-2 (TRCN0000297123), sh-3 (TRCN0000277891), sh-4 (TRCN0000277939) and sh-5 (TRCN0000297059), and a nontargeting shRNA (SHC002) (scramble) were used. All vectors were purchased from the Broad Institute MISSION shRNA library (Sigma, St. Louis, MO, USA).

BxPC-3 and Capan-1 cells were transduced with these vectors by lentiviral infection following the protocols previously described [61]. For the selection of the cells that contained the vector pLKO.1-puro, a dose response curve for antibiotic selection (kill curve) was performed. Resistant cells were selected by adding puromycin dihydrochloride (Sigma) at a final concentration of 1.5 µg/mL for BxPC-3 and 1 µg/mL for Capan-1 cells.

### 4.4. Reverse Transcription and Quantitative Real-Time PCR (RT-qPCR)

RNA extraction was performed using the RNeasy^®^ Mini kit (Qiagen, Hilden, Germany). RNA columns were treated with RNAse-Free DNase I. Eluted RNA yield and purity were determined using a NanoDrop™ instrument (ND-1000, Thermo Fisher Inc., Waltham, MA, USA). Of the total RNA 2 µg was reverse transcribed to single-stranded cDNA using MultiScribe™ Reverse Transcriptase and random hexamer primers (Applied Biosystems Inc., Foster City, CA, USA). Quantification was performed as previously described [10] with TaqMan Pre-Designed Gene Expression Assays™. Primers used for the evaluation of each glycosyltransferase and the housekeeping gene: ST3GAL3 (Hs00544033_m1), ST3GAL4 (Hs00920871_m1), ST3GAL6 (Hs00196085_m1), FUT3 (Hs01868572_s1), FUT5 (Hs00704908_s1), FUT7 (Hs00237083_m1) and TBP (Hs99999910_m1). The results were analyzed by the delta–delta Ct method and using the TATA-box binding protein (TBP) gene as a reference for calculation. Three technical replicates were performed for each sample and gene. At least three independent assays for the expression gene analysis of KD cell lines were performed. Results were expressed as mean ± SD.

### 4.5. Flow Cytometry Analysis

Detection of sialic acid determinants was performed by indirect fluorescence as previously described [9]. Briefly, 1 × 10^5^ viable cells were incubated in the presence (or absence, for negative controls) of the corresponding primary antibodies or biotinylated lectins. After washing, cells were incubated with the correspondent secondary antibody or with streptavidin conjugated to Alexa Fluor 488 (Invitrogen, Carlsbad, CA, USA). Next, the median and geomean fluorescence of the cells was determined using a FACSCalibur flow cytometer (Becton Dickinson Immunocytometry Systems, San Jose, CA, USA) equipped with CellQuest™ software (Becton Dickinson). At least three independent assays for each sample were performed. To express the percentage of decrease or increase of sialylated glycan determinants in KD cells vs. the corresponding scramble cells, first the ratio of the median values of each sialylated determinant of the KD cells vs. scramble cells was determined for each experiment, and then the mean of these ratios of the three experiments (ratio mean) was calculated and the relative percentage change was expressed as: (1-ratio mean) ×100.

### 4.6. E-selectin Adhesion Assays

Adhesion of BxPC-3 and Capan-1 cells to E-selectin was assessed as previously described [62] with minor modifications: 96-well maxisorp microplates (ThermoFisher) were coated for 24 h with 5 μg/mL of rhE-selectin (R&D Systems, Minneapolis, MN, USA) in PBS or PBS 1% BSA (as negative control). Wells were aspirated and then blocked with PBS 1% BSA. Next, 1 × 10^5^ BxPC-3 or Capan-1 cells were added and allowed to settle for 1 h at 37 °C in the incubator. In selected experiments, cells were previously incubated with anti-sialyl-Lewis x mAb. Plates were then gentle washed with PBS and the remaining adherent cells were estimated with a proliferation assay (MTS, Promega, Madison, WI, USA) following the manufacturer’s instruction. For flow-based assay, a flow chamber of PDMS was coated with rhE-selectin/CD62E Fc Chimera (R&D) at 10 µg/mL after incubation with goat anti-Human IgG Antibody, Fc, FITC conjugated (Millipore) at a final concentration of 10 µg/mL. Once incubated, devices were blocked with a solution of PBS 1% BSA for 1 h at RT. PDA cells (BxPC-3 and Capan-1) were resuspended at 3 × 10^5^ and 2.5 × 10^5^ cells/mL respectively in PBS 0.1% BSA. Cells were perfused over the immobilized E-selectin-coated devices at a wall shear stress level of 1.1 dyn/cm^2^, using a parallel plate flow chamber as previously described [43,63]. The total number of binding events in a single field of view during a 2-min period was recorded and quantified with a phase contrast 10 × Ph1 objective of a Nikon Inverted microscope.

### 4.7. Transwell Migration Assay

Cell migration was evaluated using modified Boyden chambers in 24 well plates as previously described [10]. Briefly, cells were grown in absence of FBS for 24 h before they were harvested. Prior to cell seeding, serum free medium with 0.001% of collagen type 1 from calf skin (Sigma) was placed in the upper part of the 8 μm pore size ThinCerts TM inserts (Greiner bio-one, Kremsmünster Austria). Detached cells were resuspended in PBS 1% BSA. In selected experiments, cells were previously incubated 20 min with anti-sialyl Lewis X mAb diluted 1:10 (BD Biosciences). The cell inserts were seeded with 3.5 × 10^4^ cells for Capan-1 and 2.5 × 10^4^ for BxPC-3 in the top chamber. Cells were let to migrate for 18 h at 37 °C. After that, inserts were washed, fixed and stained. Non-migrated cells were removed from the top surface of the insert using cotton swabs. Then at least 20 random camps were photographed at 10× magnification under the microscope with a CKX41 inverted microscope (Olympus Optical Co., Ltd., Tokyo, Japan).

### 4.8. Polydimethylsiloxane (PDMS)-Based Microchannel Migration Assay

PDMS-based microchannels devices were fabricated as previously described [64]. Devices were coated with 20 μg/mL of collagen type 1 to facilitate cell adhesion. Cell migration was visualized and recorded via time-lapse live microscopy (Nikon) at 37 °C in a humidified atmosphere of 5% CO_2_. Phase contrast time-lapse images were taken for 24 h in a 10 min interval with a 10× Ph1 objective. The spatial x and y positions of all non-dividing and viable cells that entered and migrated in the microchannels were tracked overtime with the Manual Tracking plugin in ImageJ. Motility parameters, namely velocity, speed and persistence, were computed using a custom-written MATLAB code as previously described [41,42].

### 4.9. Transwell Invasion Assay

Cell invasion was evaluated using modified Boyden chambers in 24 well plates. Prior to starved-FBS cell seeding, chambers were precoated with 25 µg of Matrigel (Corning, Corning, NY, USA). Then, the mixture was allowed to polymerize at 37 °C. After trypsinization, detached cells were washed and resuspended in PBS 1% BSA. In selected experiments, cells were previously incubated 20 min with anti-sialyl Lewis X mAb diluted 1:10 (BD Biosciences). Then, 5 × 10^4^ cells for Capan-1 and 4 × 10^4^ for BxPC-3 were seeded in the top chamber. After 30 min, medium containing 10% FBS for BxPC-3 or 20%FBS for Capan-1 cells was added to the bottom as chemoattractant. Cells were let to invade for 24 h at 37 °C, and after that inserts were washed, fixed and stained. Non-invaded cells were removed from the top surface. Then, at least 20 random camps were photographed at 10× magnification under the CKX41 inverted microscope (Olympus).

### 4.10. Statistical Analysis

All data are presented as the mean ± standard error of the mean (SEM) or standard deviation (SD) from 3 independent experiments, unless otherwise stated. In the analyses of the sialylated determinants expression of the cells’ panel (Figure 1B), two values were used for Capan-2, HPAF-II, Panc 10.05 and SW 1990. Statistical significance was determined between pairs of data with a *t*-test, or between groups of data with one-way ANOVA and a Tukey’s multiple comparison post-hoc test. Dunnett’s multiple comparisons test was used to compare each of the shRNAs to scramble in migration microdevices. Figures were designed with Prism7-GraphPad.

## Figures and Tables

**Table 1:** Percentage of decrease (↓) or increase (↑) of sialylated glycan determinants in BxPC-3 KD cells vs. the corresponding control (scramble, SC) cells determined by flow cytometry.

Cells	% change in sLe^x^	Sig.^1^	Sig.^1^	% change in sLe^a^	Sig.^1^	% change in α2,6-SA	Sig.^1^	Sig.^1^
	vs. SC cells ^1^	vs. shST3GAL4_1	vs. shST3GAL3_7	vs. SC cells ^1^	vs. shST3GAL3_7	vs. SC cells ^1^	vs. shST3GAL4_1	vs. shST3GAL3_7
shST3GAL4_1 BxPC-3	↓68% (***)	-		↑30%^2^		↑53% (***)	-	
shST3GAL3_7 BxPC-3	↓33% (***)	***	-	↓2% (ns)		↑21% (ns)	*	-
shST3GAL3_9 BxPC-3	↓37% (***)	***	ns	↓34% (*)	*	↑41% (**)	ns	ns

**^1^** ANOVA and Tukey’s multiple comparison post-hoc test was performed. ns: not significant; *p* < 0.05: *; *p* < 0.01: ** and *p* < 0.001:***. **^2^** Determined by CA19.9 immunoassay.

**Table 2:** Percentage of decrease (↓) or increase (↑) of sialylated glycan determinants in Capan-1 KD cells vs. the corresponding control (scramble) cells determined by flow cytometry.

Cells	% change in sLe^x^	Sig.^1^	Sig.^1^	Sig.^1^	% change in sLe^a^	Sig.^1^	Sig.^1^	Sig.^1^	% change in α2,6-SA	Sig.^1^	Sig.^1^	Sig.^1^
	vs. SC cells ^1^	vs. shST3GAL4_1	vs. shST3GAL4_4	vs. shST3GAL3_7	vs. SC cells ^1^	vs. shST3GAL4_1	vs. shST3GAL4_4	vs. shST3GAL3_7	vs. SC cells ^1^	vs. shST3GAL4_1	vs. shST3GAL4_4	vs. shST3GAL3_7
shST3GAL4_1 Capan-1	↓64% (***)	-			↓32% (*)	-			↑35% (**)	-		
shST3GAL4_4 Capan-1	↓63% (***)	ns	-		↓45 (**)	ns	-		↑24% (*)	ns	-	
shST3GAL3_7 Capan-1	↓61% (***)	ns	ns	-	↓43% (**)	ns	ns	-	↑20% (ns)	ns	ns	-
shST3GAL3_9 Capan-1	↓73% (***)	ns	ns	ns	↓52% (***)	ns	ns	ns	↑12% (ns)	*	ns	ns

**^1^** ANOVA and Tukey’s multiple comparison post-hoc test was performed. ns: not significant; *p* < 0.05: *; *p* < 0.01: ** and *p* < 0.001:***.

## Abbreviations

KD	knockdown
mAb	monoclonal antibody
PDA	pancreatic ductal adenocarcinoma
SA	sialic acid
SC	scramble
sLe	sialyl-Lewis
sLe^x^	sialyl-Lewis x
sLe^a^	sialyl-Lewis a
ST	sialyltransferase
SD	standard deviation
SEM	standard error of the mean
WB	western blot
